# Small-Sided-Game-Induced Mechanical Load in Adolescent Soccer: The Need for Care and Consideration for Athlete Preservation

**DOI:** 10.1177/19417381241296063

**Published:** 2024-11-22

**Authors:** Jamie Salter

**Affiliations:** † InSPIRe Group, School of Science, Technology and Health, York St John University, York, UK

**Keywords:** mechanical load, adolescent, small-sided games, maturation, injury

## Abstract

**Context:**

The logistical efficiency and flexibility of small-sided games (SSG) to develop various soccer-specific attributes simultaneously make them a staple component of contemporary training programs in youth soccer. Their high ecological validity and consequential high utilization mean that if not considerately prescribed, players may be exposed to frequent repetitive mechanical stress that may induce maladaptation in skeletally and/or load-naïve or sensitive athletes. The purpose of this clinical review is to summarize mechanical load adaptations associated with the manipulation of area per player in SSG to outline the mechanistic pathway of load-related injuries in skeletally maturing athletes and to offer practical guidelines for coaches for the preservation of athlete health.

**Evidence Acquisition:**

A nonsystematic search of computerized databases of peer-reviewed articles in English between 2010 and the present was used, and a critical appraisal of existing literature was subsequently conducted.

**Study Design:**

Clinical review.

**Level of Evidence:**

Level 4.

**Results:**

The temporary relative strength deficit and inefficiency of the musculotendinous system associated with accelerated growth increase the mechanical cost of activity. As a result, the load tolerance (ie, tolerant, naïve, or sensitive) of athletes is transiently reduced as the musculoskeletal system struggles to attenuate force absorption adequately. Repeated exposure to submaximal mechanical loads that stimulate the accumulation of “microdamage” in structural tissue may lead to aggravation and/or tissue failure at connective sites in skeletally fragile athletes.

**Conclusion:**

Coaches and practitioners need to individualize exposure to mechanical load for load-tolerant, naïve, and sensitive athletes during adolescence. Subtle changes to SSG prescription including modifying the area per player, inclusion of goalkeepers, constrained floaters, and management of work; rest ratios can offer practical and efficient methods to mitigate risk without derailing the development process. This, in turn, should contribute to reducing injury burden in this population and enhance developmental opportunities for young players.

**Strength of Recommendations:**

A. Recommendation based on consistent and good-quality evidence published from 2010 onwards.

From midadolescence, competitive soccer matches are typically played with 2 teams of 11 players in a playing area between 82 × 50 m and 91 × 65 m.^51^ It is common for coaches and practitioners to manipulate the area per player (ApP, expressed in square meters) during training practices as an efficient training modality to enhance technical, tactical, psychological, and physical characteristics simultaneously.^3,9,22,46^ Extensive research has explored the techno-tactical and the psycho-physiological impact that manipulating area per player can have on player development.^1,3,8,9,10,13,16,31,37^ This literature offers valuable practical guidance for coaches targeting specific characteristics in a soccer-specific manner (ie, enhance aerobic endurance or exposure to high-speed running). Manipulating the ApP through small-sided games (SSG) has also been shown to be effective for coaches in identifying, selecting, and developing talented young players.^17,63^ Studies indicate that reducing the number of players per team increases the individual technical actions (ie, touches, dribbles, and passes),^4,11,12^ which naturally enhances the opportunities for technical and tactical (ie, decision-making) development for young players.

The logistical simplicity, flexibility, and efficiency of SSG mean they are a valuable and versatile modality for coaches and practitioners working with youth soccer players, and, as a result, they are a staple component of contemporary training prescription. However, evidence suggests that reducing the ApP may increase the frequency of high-intensity actions (ie, accelerations, decelerations, changes of direction and short sprints),^10,23,36,39^ which may influence fatigue response and extend recovery time as a result of the propulsive and braking forces associated with these movements.^36,39^ These mechanical stress-inducing activities impact the musculoskeletal system (ie, cartilage, bone, ligaments, muscle, and tendon) and are related directly to tissue damage and repair, with only a narrow window of exposure considered “optimal.”^60^ Although effective in many ways, frequent exposure to mechanically demanding components of SSG without adequate recovery between sessions needs careful consideration in adolescent athletes. There is a substantial increase in growth, overuse, and general noncontact-related injury incidence during adolescence, which may be attributed to frequent exposure to low-to-moderate mechanical loads.^56,61^ Therefore, the purpose of this clinical review is to summarize the physiological, but primarily mechanical, responses associated with the manipulation of ApP, and to explore the possible mechanistic pathway of load-related injuries in developing athletes, to offer practical guidelines for coaches for the preservation of athlete health.

## Load Characteristics of SSG

The flexibility of SSG to expose players to situations or environments to develop specific attributes of performance is connected to an array of variables that can be manipulated by the coach. These include pitch dimension, number of players, numerical balance, the inclusion of goalkeepers and/or goals, game duration, work:rest intervals, rule selection, and coach encouragement.^22^ Coaches may wish to either under- or overload the physiological and/or mechanical demands of SSGs, and consequently manipulate these variables to suit their periodized objectives.^32^ From a physiological or metabolic standpoint, evidence implies that larger areas (>100 m^2^ ApP) typically stimulate higher overall intensities; verified by greater blood lactate response, higher heartrates, elevated perceived intensities, greater overall and high-speed distances, and higher peak velocities.^8,9,22,43,46^ Therefore, larger ApP formats may be more useful when developing posterior chain activity due to the increased high-speed running and sprint distances observed in these formats. Unsurprisingly, ApP that more closely replicates competitive dimensions (250-350 m^2^) is more representative of match demands and therefore can be used to develop and maintain match fitness in various age groups.^8,46^ These findings remain consistent even when additional variables are manipulated and, although responses may be moderated,^9,16,22,45,47,50,53^ they follow the same physiological pattern.

From a mechanical perspective, the opposite would appear to be true. Accelerometery-based variables (ie, accelerations, decelerations, and changes in direction) often achieve similar values to peak periods of official matches in high-density SSG.^14^ Ispirlidis^23^ compared various physical parameters between small (2.4 m^2^) and large (150 m^2^) ApP, identifying significantly more medium-high-intensity accelerations and medium-intensity decelerations in the smaller ApP condition. This is supported by Guadino et al,^19^ who reported more frequent changes in velocity, and increased moderate accelerations and decelerations with smaller ApP conditions (75 m^2^ vs 98 m^2^ or 135 m^2^). In addition, Lacombe et al^32^ outlined the relatively higher mechanical work in smaller ApP conditions, particularly when applied for short work durations (~2 minutes). The constitution of rapid changes in velocity and direction elicits high mechanical loads due to the propulsive and braking forces involved.^60^ Eccentric work combined with lateral and/or anterior foot placement is required to decelerate the body, with large concentric work and utilization of the stretch-shortening cycle (SSC) required thereafter to accelerate in a new direction.^26,36,54^ This chain of events may explain the more intense and prolonged exercise-induced muscle damage response observed after high-density ApP (62.5 m^2^) SSG compared with low-density (284 m^2^) SSG observed in soccer players.^39^ Players reported significantly higher levels of self-reported delayed onset muscle soreness in knee extensors and flexors and concomitant elevated creatine kinase levels between 24 and 72 hours post-SSG intervention.

The findings presented above illustrate that the design, prescription, and delivery of SSG significantly influence the acute response and, possibly, the subsequent chronic adaptations experienced by players, and that care and consideration are required to preserve athlete health. As reported in adult soccer, there is a need to include a diverse selection of training modalities (ie, intensive SSG, extensive SSG, high-speed running, eccentric training) for optimal holistic development of young players, that is periodized within- and between microcycles.^15,21,38^ Although likely variable between talent development environments, the logistical and situational constraints facing practitioners and coaches may constrain the format and density of SSG (eg, pitch hire costs, space and goal availability, squad size, club/academy philosophy, and playing style). Therefore, practitioners must be aware of the various ways in which they can manipulate the outlined variables to mitigate risk and prevent excessive fatigue while exposing athletes to adequate, varied, and appropriate physiological and mechanical loads.

## Influence of Growth and Maturation

The adolescent growth spurt (often referred to as the period surrounding peak height velocity [PHV]) produces a unique scenario whereby developing athletes experience myriad naturally occurring physical adaptations that may temporarily predispose them to elevated injury risk.^56^ Although the physical changes that occur are uniform (eg, increased limb length, increased muscle mass, enhanced SSC function, tendon stiffness, and motor unit recruitment) the timing, tempo, and magnitude of these are highly variable between players.^41,42,57^ Biological age can vary between 4 and 6 years (9.6-14.1 years) in chronological age groups, with differences in height (~17%), mass (~50%), and fat-free mass (~21%) being substantial.^18,49^ The physical growth of the skeletal structures stimulates a mechanotransductive response from connective and soft tissues, resulting in a transient period of relative strength deficit that impedes coordination, movement, and general athleticism (often referred to as adolescent awkwardness).^33,41^ This period exposes athletes to an elevated risk of traction apophyseal injuries due to a transient deficit in bone mineralization, increased bone porosity,^34^ and increased stress on connective tissue, even in relaxed states (referred to as tissue preload) due to the musculoskeletal imbalances created.^44^ Calculations have predicted that, during this period, lower-limb muscles must develop ~30% more force to produce the same relative acceleration,^59^ and that less mature players perceive training sessions to be significantly more intense than more mature counterparts.^49^

The rapidly evolving musculoskeletal composition and the relative disproportionate changes in limb and trunk length create a notion of “skeletal fragility.” This fragility is considered a significant contributor to the increase in injury burden during (57.9 days) and post-PHV (89.4 days) compared with pre-PHV (44.6 days).^6,58^ Studies report that 12% to 45% of all injuries were growth-related and that many (46% to 72%) were noncontact and moderate in severity (30% to 43%).^35,48,55,62^ Based on this, the mechanical load-adaptation pathway of training and competition is adversely affecting many adolescent athletes, with each severe injury being reported to reduce overall development time by approximately 10%, therefore affecting long-term outcomes.^27^ This may well be a byproduct of the naturally occurring process of maturation, but is likely exacerbated by development pathway guidelines prescribing age-related intensification in training time and/or frequency that directly align with key developmental stages.^40,52^

The Elite Player Performance Plan (EPPP) governs UK soccer academies and prescribes staff, facility infrastructure, and coaching exposure criteria that academies must meet to maintain their audited categorization.^40^ This criteria-driven process influences the development environment of each academy and includes systemic age-related increments in provision (ie, coaching hours) according to development stage (ie, Foundation, Youth Development or Professional Development Phase), rather than biological age. Although logical as the advanced training age allows players, in theory, to tolerate training demands better, it will likely increase their exposure to SSG, and subsequently mechanical loads. For some athletes, this may present minimal issues and facilitate continual development; however, for many, this progressive and age-informed increment in load coincides with the period of skeletal fragility and may lead to structural failures in the form of chronic, growth and/or overuse-related complaints that escalate into injury.^60^ Unfortunately, in most cases, the onset of such issues is difficult to detect and relies primarily on retrospective diagnosis only once lagging indicators (ie, pain and/or discomfort or inflammation) present themselves.

## Mechanical Load-Adaptation Pathways in Adolescent Players

Biological tissue (eg, bone, muscle, tendon) failure occurs when the strength of the material is surpassed by excessive stress (ie, force per unit of area) and strain (ie, amount of deformation) induced by the application of force, either singular high-magnitude or repeated lower-magnitude loads.^28,29^ Theory indicates that repeated submaximal loading causes the accumulation of ‘micro-damage’ in structural tissue, and when the rate of accumulation exceeds the rate of biological repair, injury, or failure occurs.^64^ Thresholds of tissue failure vary between adult and adolescent athletes as repetitive forces applied to an immature skeleton cause aggravation and overuse-related complaints at vulnerable sites (ie, apophyses).^30,34^ Jayanthi et al^24^ argue that systematic increments in training load exposure are possible and should be encouraged in youth athletes, but recognize variations in load tolerance and thus categorize youth athletes as either (1) load tolerant, (2) load naïve, or (3) load sensitive. Load-tolerant athletes have typically passed PHV (>96% percentage of predicted adult height [PPAH]), have manageable weekly workloads in relation to their age (hours < age) and chronic exposure, limited previous injuries, and have low levels of sport specialisation.^25^ Load-naïve athletes may have relatively high acute loads compared with chronic exposure (ACWR > 1.5), be approaching PHV (~85% PPAH), have suboptimal training and competition ratios (<1:1), and a degree of sport specialization. Load-sensitive athletes are typically the most at risk and are characterized by those experiencing PHV (85% to 96% PPAH), high relative workloads (hours > age; ACWR > 2.0), and highly sport-specialized and with suspected overuse injury symptoms.^25^ As a result of biological diversity, it is likely that, in a squad of players, coaches will have a mixture of load-tolerant, naïve, and sensitive athletes, and will need to consider each player’s suitability for prescribed practices; thus, an individualized approach is required.

Frequent exposure to the low-to-moderate intensity mechanical loads typically produced by small ApP practices may be a contributing factor to the high prevalence of growth/overuse injuries in adolescent populations, at least for load-naïve or sensitive athletes. A theoretical mechanistic-causal pathway to explain this notion has been presented recently by Kalkhoven,^28^ who suggested that the repetitive mechanical loads elicited by frequent exposure to low-to-moderate mechanical loads (eg, reduced ApP SSG) will gradually fatigue a tissue until a critical damage threshold is exceeded. The mechanical strength of a tissue is considered time-varying as it deteriorates progressively over time owing to the cycling loading, which can be accelerated in athletes who are “skeletally fragile,” have minimal recovery time, and are exposed to age, rather than biological-related, intensification of loading (eg, due to EPPP requirements). The transient changes outlined above related to maturation (i.e., deficit in bone mineralization, increased bone porosity, and tissue preload) result in a reduced critical damage threshold, and thus may partially explain the increased injury incidence at this time.^35,48,61^ The frequent changes in direction and velocity require rapid activation of the SSC and co-contraction of major muscle groups to maintain the structural integrity of joints, both of which are influenced substantially by maturation.^42^ There is a gradual increase in SSC function with biological age, attributed to various factors, including increased motor-unit (particularly Type II motor-unit) recruitment, contraction speeds, preactivation, pennation angle, rate of force development and cross-sectional area.^41,42^ Combined with suboptimal SSC function, the relative strength deficit imposed by immature muscular development on a heavy skeletal frame elevates the relative mechanical cost of even submaximal activities, reducing the athletes ‘ceiling’ (ie, critical damage threshold).^24^ As a result, the load tolerance of athletes is reduced as the musculoskeletal system struggles to attenuate force absorption adequately. Estimates examining the nonlinear relationship between load, magnitude, and muscle damage suggest that reducing imposed stressors by 10% generally yields a corresponding 100% increase, or more, in the number of cycles to failure.^29^ Thus, we are recommending that only small modifications in SSG prescription for load-naïve and sensitive athletes are required, which may mean they can continue to train and compete as prescribed by policy without experiencing adverse mechanical load adaptations/injury.

## Practical Applications

**Figure 1.**
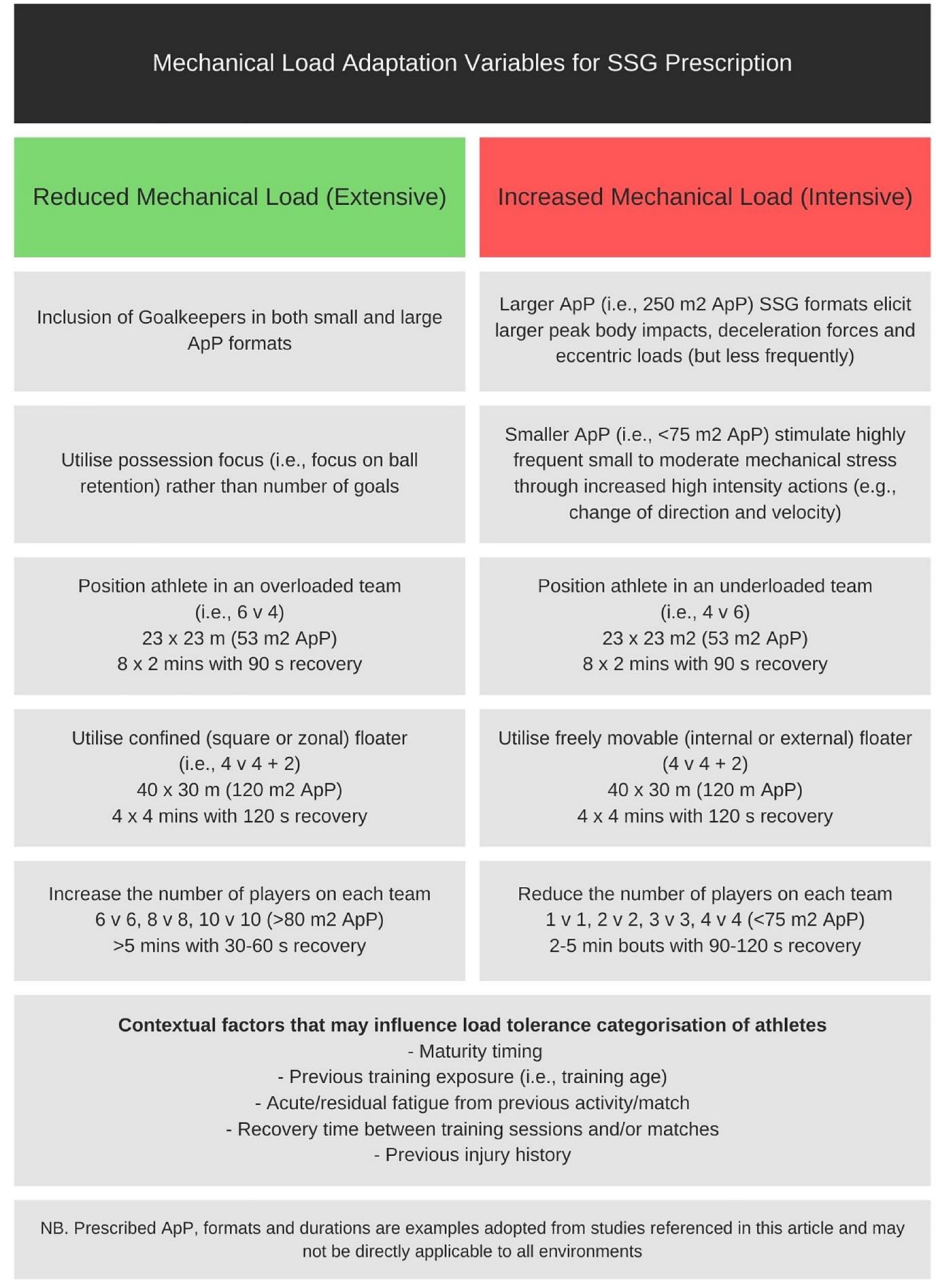
Guidance on variables to manipulate mechanical load-adaptation pathways in SSG activities. ApP, area per player; SSG, small-sided game.

It is important to clarify that the author is not suggesting that utilizing SSG or high-density ApP activities is detrimental to adolescent players. In contrast, the author believes SSG to be a highly effective, efficient, and pragmatic way of identifying, developing, and monitoring adolescent players throughout biological maturation.^17,63^ However, the focus of this clinical review is to highlight the potential consequences of a poorly considered, or “one size fits all” prescription of a fundamental modality of player development, and to promote proactive and cognisant prescription of this to help reduce growth-/overuse-related injury burden. Therefore, the final section outlines some constraints and variable manipulations that might offer coaches practical, efficient, and effective ways to preserve load-naïve or sensitive athletes (Figure 1).

There is a consensus in the literature that lower-density ApP activities (ie, extensive) increase the physiological load via increased distances covered in various speed thresholds, maximum velocities achieved and overall metabolic load.^9^ Therefore, these larger area sizes may elicit larger peak deceleration forces, higher peak eccentric forces (particularly on the posterior chain) and higher body impacts, but at lower frequencies compared with high-density ApP.^7,19,20^ Therefore, utilizing SSG formats with an extensive focus would reduce the overall mechanical load (due to less frequent actions), while also exposing athletes to other critical performance attributes required for their development (ie, metabolic stress, maximal velocity running). In addition, Lacome et al^32^ concluded that decreasing the number of players per team increased the high-intensity actions and changes in velocity (ie, accelerations and decelerations) and, therefore, teams with more players reduce the frequency of high-intensity involvements, which may, in turn, lessen the overall load of the SSG. They also suggested that high mechanical load from high-density SSG was sustainable only for short SSG bouts (<5 minutes) with longer rest periods (ie, 90-120 seconds). Therefore, although paradoxical in theory, another strategy to minimize the mechanical load is to prescribe longer, or continuous, bouts with shorter recovery periods—a notion that Branquinho et al^5^ support. Therefore, coaches looking to prescribe sessions with reduced mechanical load should strive for larger ApP (>250 m^2^), include goalkeepers, have a focus on possession, and increase the number of players per team (ie, >6 vs 6). Contrastingly, if practitioners wish to prescribe sessions with high-mechanical loads (ie, intensive) they would utilize smaller ApP (<75 m^2^), have no goalkeepers, and use small teams (ie, <4 vs 4). Ideally, at least 1 exposure to both formats across the microcycle would provide adequate load prescription for most players; however, if logistical constraints prevent this, coaches should incorporate as many of the desired variables as possible when implementing SSG sessions.

As previously outlined, there is a likelihood that teams will be comprised of players with varying load tolerance levels and biological diversity. Therefore, practitioners may need to utilize some of the confined area and/or mismatched team constraints to adequately load those players differently within the same session. For example, Guard et al^20^ compared the load profiles of players in unbalanced teams and observed elevated metabolic and mechanical loads in players on teams with an inferior number of players (ie, 4 vs 6). Therefore, this offers practitioners a useful option to subtly reduce the stress imposed on naïve or sensitive athletes, by placing them on an overloaded team for longer durations, while challenging load-tolerant athletes on underloaded teams more so. Also, Asian-Clemente et al^2^ suggest that including players as floaters may be a useful strategy to minimize mechanical stress, and that modifying their involvement to either internal, external, or zonal floaters can all incrementally reduce loads. Therefore, load-naïve or sensitive players may be utilized as floaters or confined to zones for a greater proportion of the session than load-tolerant athletes, as a method of individually managing their exposure. The simple manipulation of overloads and floater formats is a logistically simple tool that can be utilized by practitioners “on-the-fly” as they monitor the session but requires previous appreciation and awareness of those in the various load tolerance groups for effective application.

## Summary

The logistical efficiency and flexibility of SSG to develop various attributes simultaneously mean they are a staple component of contemporary training programs for youth soccer coaches. The host of manipulative variables available makes the SSG format pliable to accentuate the development of various technical, tactical, or physical properties. The frequency of their prescription in adolescent football (ie, 1-3 times per week) means that, if not considerately prescribed, players will be exposed to overly frequent repetitive mechanical stress that may induce maladaptive load-adaptations in skeletally and load-naïve or sensitive athletes. Therefore, coaches need to embrace subtle variations in rules, format, team configuration, and duration to optimize the load response and preserve athlete health. These modifications may be discrete and athlete-specific or applied to whole groups as required (eg, biobanded). They should then be reviewed regularly in conjunction with specialist support staff through longitudinal monitoring of biological maturation and injury symptoms.
